# Challenges to Getting Older Adults Geriatric Care: Lessons From a National Rural Geriatrics Program

**DOI:** 10.1093/geroni/igaf122.1227

**Published:** 2025-12-31

**Authors:** Lauren Moo, Eileen Dryden

## Abstract

Rural older adults have higher rates of chronic diseases and have more complex care needs than their urban counterparts. Interdisciplinary geriatrics teams have the necessary expertise to meet these complex needs but access to geriatric care in rural areas is challenging. It is critical to address the needs of aging rural older adults, so that many can successfully age in place. Information on regional needs of the older adult population and availability of existing resources is critical to informing efforts to reach older rural Veterans and the appropriate mechanisms to support these efforts. Within the Veteran healthcare system, the GRECC Connect program has improved access to geriatric care for older rural veterans using telehealth strategies while providing support to rural frontline teams as they care for complex older adults and their families. While current steps to ensure sustainment of these services are underway, we share the lessons learned on developing geriatric care services in rural areas-- highlighting the limited access to geriatric care in rural areas, developing and advocating for developing geriatric care services in resource poor areas, potential and limitations of a hub and spoke model for geriatric care and barriers and successes in sustaining and integrating geriatric care services to serve rural areas.

